# Declines in Connected Language Are Associated with Very Early Mild Cognitive Impairment: Results from the Wisconsin Registry for Alzheimer’s Prevention

**DOI:** 10.3389/fnagi.2017.00437

**Published:** 2018-01-09

**Authors:** Kimberly D. Mueller, Rebecca L. Koscik, Bruce P. Hermann, Sterling C. Johnson, Lyn S. Turkstra

**Affiliations:** ^1^Wisconsin Alzheimer’s Institute, University of Wisconsin School of Medicine and Public Health, Madison, WI, United States; ^2^Department of Neurology, University of Wisconsin School of Medicine and Public Health, Madison, WI, United States; ^3^Geriatric Research Education and Clinical Center, William S. Middleton Memorial Veterans Hospital, Madison, WI, United States; ^4^Wisconsin Alzheimer’s Disease Research Center, University of Wisconsin School of Medicine and Public Health, Madison, WI, United States; ^5^Department of Communication Sciences and Disorders, University of Wisconsin–Madison, Madison, WI, United States; ^6^Neuroscience Training Program and Department of Surgery, University of Wisconsin–Madison, Madison, WI, United States

**Keywords:** Alzheimer’s disease (AD), mild cognitive impairment (MCI), language, speech, connected speech, discourse analysis, picture description, verbal fluency

## Abstract

Changes to everyday spoken language (“connected language”) are evident in persons with AD dementia, yet little is known about when these changes are first detectable on the continuum of cognitive decline. The aim of this study was to determine if participants with very early, subclinical memory declines were also showing declines in connected language. We analyzed connected language samples obtained from a simple picture description task at two time points in 264 participants from the Wisconsin Registry for Alzheimer’s Prevention (WRAP). In parallel, participants were classified as either “Cognitively Healthy” or “Early Mild Cognitive Impairment” based on longitudinal neuropsychological test performance. Linear mixed effects models were used to analyze language parameters that were extracted from the connected language samples using automated feature extraction. Participants with eMCI status declined faster in features of speech fluency and semantic content than those who were cognitively stable. Measures of lexical diversity and grammatical complexity were not associated with eMCI status in this group. These findings provide novel insights about the relationship between cognitive decline and everyday language, using a quick, inexpensive, and performance-based method.

## Introduction

There have been significant advances in detecting neuropathological changes early in the Alzheimer’s disease (AD) continuum, and these have spurred the development of multiple clinical trials, investigating both pharmacological and non-pharmacological early interventions. As a result, there is an urgent need for sensitive tools that measure cognitive and functional change at very early stages of disease (Snyder et al., 2014). One candidate tool is analysis of connected language in discourse, which has shown to be an informative measure of language problems across mild to moderate stages on the AD continuum (Nicholas et al., 1985; Bayles et al., 1992; Tomoeda and Bayles, 1993). “Connected language” (also referred to as “connected speech,” “spontaneous speech,” or “discourse”) refers to spoken language that is used in a continuous sequence, as in everyday conversations. The production of spontaneous speech involves the use and coordination of multiple cognitive and physiological processes, including retrieval from semantic and episodic memory, the ability to sustain and divide attention for error monitoring, and the reliance upon working memory for syntax production (Carter et al., 1998; Hartsuiker and Barkhuysen, 2006; Barbeau et al., 2012). As such, connected language analysis may be sensitive to early cognitive changes, and also may yield performance-based measures that are more representative of the actual skills needed for activities of daily living than are typical standardized language and other cognitive tests. Few studies have examined the earliest point at which connected language changes are observable [i.e., the Mild Cognitive Impairment (MCI) phase or before], and the literature that exists has significant limitations. Additionally, there are few longitudinal studies that have documented whether or not subtle changes in discourse can be reliably measured over time. Longitudinal data are important because both discourse features and the clinical expression of MCI and AD are heterogeneous (Duong et al., 2005), so measurement of within-person change over time is critical for characterizing change. Thus, there is a need for both studies of connected language in persons with early cognitive decline, and also studies that examine performance over time.

The few studies of connected language in MCI have shown similar deficiencies in semantic content and semantic processing as in AD dementia. The earliest prospective evidence of connected language change in true preclinical AD came from Cuetos et al. (2007), who compared connected language samples from a group of 19 asymptomatic individuals carrying the hereditary mutation of the *Presenilin-1* gene, to similar-aged family members without the mutation, prior to evidence of significant cognitive decline. They found that mutation carriers expressed less semantic content in the preclinical phase of AD (mean age = 43) than non-carriers (Cuetos et al., 2007). Retrospective studies of novelist Iris Murdoch’s writings indicated that impoverished vocabulary and less complex syntax were evident in her 40 s, decades before her clinical diagnosis of AD (Le et al., 2011). Similarly, Berisha et al. (2015) retrospectively analyzed unscripted speeches of former U.S. President Ronald Reagan and found increases in filled pauses and decreases in unique words over time, well in advance of his diagnosis of AD. Ahmed et al. (2013b) retrospectively analyzed picture descriptions of nine autopsy-confirmed patients with AD at three stages of disease (MCI, Mild AD, Moderate AD), and detected a progressive decline in efficiency (time required to express ideas) and semantic content.

Although these findings and others (Tomoeda et al., 1996; Bschor et al., 2001; Fleming and Harris, 2008; Choi, 2009; Drummond et al., 2015) offer encouraging evidence for using connected language as a measure of change in AD, existing studies have significant limitations. First, studies either had small sample sizes or were single-case studies, limiting generalizability to other cohorts. Second, in the few studies with larger sample sizes (Forbes-McKay et al., 2013; Fraser et al., 2016), patients were in the mild to moderate stages of AD and results may not generalize to earlier stages of disease when compensatory abilities can result in more subtle impairments in functional language. Third, in the case of retrospective analyses (Garrard et al., 2005; Ahmed et al., 2013b), standardization procedures often were not reported so studies may be difficult to replicate. Fourth, the potential effects of psychological and biological factors (e.g., depression, anxiety, sleep) on connected language are not well studied, and the contribution of these potentially modifiable factors to discourse variability is unknown. In order to determine the feasibility and sensitivity of connected language analysis and its potential sources of variability, particularly for the purposes of assessment and monitoring within clinical trials, larger-scale longitudinal studies of preclinical populations are necessary.

The present study investigated connected language over time in a late middle-aged cohort enriched for risk of sporadic AD. The Wisconsin Registry for Alzheimer’s Prevention (WRAP) is an ongoing longitudinal study that began in 2001, with an objective of identifying cognitive and biomarker profiles that may be predictors of AD risk. In addition to completing an extensive neuropsychological test battery at study visits every 2 years, participants also provide a connected language sample of a picture description task. The overarching goal of the current study was to determine whether WRAP participants who were classified with sub-clinical, very early Mild Cognitive Impairment (“eMCI”) based on standard neuropsychological tests showed evidence of cognitive decline on connected language measures. Our aim was to determine whether being classified as eMCI was associated with atypical longitudinal connected language trajectories compared to cognitive stable WRAP participants. We hypothesized that eMCI would be associated with greater decline in connected language over time.

## Materials and Methods

### Participants

#### WRAP Study Sample

The WRAP is a longitudinal study of late-middle-aged adults enriched for AD risk based on parental family history. WRAP participants are asymptomatic, English speaking, and are between the ages of 40 and 65 at the time of enrollment (Sager et al., 2005; Johnson et al., 2017). Approximately 72% of participants have a parent with either autopsy-confirmed or probable AD [family history positive (FH+)] as defined by the National Institute of Neurological and Communicative Disorders and Stroke and the Alzheimer’s disease and Related Disorders Association (NINCDS-ADRDA) criteria (McKhann et al., 1984). The sample has a higher-than-average proportion of carriers of at least one ε-4 allele of the *APOE* gene, therefore are at higher genetic risk for sporadic AD than the general population (Liu et al., 2013). Approximately 28% of participants had no parental history of AD and met the criteria that their mothers survived to at least age 75, and their fathers to at least age 70, with no dementia [family history negative (FH-)].

The WRAP study began in 2001 and currently has enrolled 1551 participants, primarily from the upper Midwest. The sample is 71% women, 88% white, and has a mean age at baseline of 54 years. The longitudinal study design includes a baseline visit, followed by a second visit 4 years later, and serial follow-up visits every 2 years thereafter. Participants are tested at one of three sites: Madison, LaCrosse, and Milwaukee, Wisconsin. WRAP participants undergo an extensive neuropsychological battery at each visit, and collection of a spontaneous language sample began at the third wave visit in 2012. This study was approved by The University of Wisconsin–Madison’s Institutional Review Board. All subjects gave written informed consent in accordance with the Declaration of Helsinki.

#### Present Study Sample

For the present study, we restricted our sample to participants who had completed language samples and neuropsychological test batteries at two time points, approximately 2 years apart (*n* = 280). Participants also had to have had a consensus diagnosis of either “cognitively healthy” (CH) or “early Mild Cognitive Impairment” (eMCI) at each of the two visits. The main component of this experimental diagnosis is a subtle cognitive deficit less pronounced than the traditional MCI diagnosis and not requiring a cognitive complaint from the participant or informant, (3) clinical MCI, (4) clinical impairment-not MCI, or (5) dementia. For additional detailed information on the consensus diagnosis process, see Koscik et al. (2016) and Johnson et al. (2017). Because the aim of this study was to capture language problems very early on in the possible-AD continuum, we excluded participants who were diagnosed with dementia, clinical MCI or “clinical impairment-not MCI” (*n* = 9). We also excluded participants who were non-native speakers of English (*n* = 2); those who had neurological diagnoses including stroke, epilepsy, Parkinson’s disease, and multiple sclerosis at either visit (*n* = 2); and those whose speech recordings were incomplete (*n* = 1); resulting in a final sample size of 264 participants (cognitively healthy = 200; eMCI = 64).

### Discourse Collection Procedure

Participants provided informed consent to have their speech recorded while describing the “Cookie Theft” picture from the Boston Diagnostic Aphasia Examination (Goodglass and Kaplan, 1983). Participants were instructed to “Tell me everything you see going on in this picture.” Evaluators provided no feedback during participants’ descriptions; however, if responses were unusually brief (e.g., one or two sentences), evaluators provided the scripted prompt, “Do you see anything else going on?” Language samples had a mean duration of 50.4 s (*SD* = 0.02), including prompts from the examiner. All responses were recorded using an Olympus VN-6200PC digital audio recorder.

### Transcriptions

Language samples were transcribed by a trained speech-language pathologist (KDM), and two trained graduate students, using Codes for Human Analysis of Transcripts (CHAT) (MacWhinney, 2000). Transcribers were blinded to the cognitive status of the participant. Utterances were segmented into C-Units, an established metric for discourse analysis defined as “an independent clause and all of its modifiers” (Hunt, 1965; Hughes et al., 1997). Transcripts were coded for automatic analyses by the Computer Language Analysis (CLAN) program (Macwhinney et al., 2011), including codes for filled and unfilled pauses, repetitions, revisions, semantic units, errors (semantic, phonological, lexical), and non-verbal behaviors (e.g., coughing, laughing). Semantic units, parts of speech, total utterances, grammatical relations, and other quantifiers were then automatically extracted by the CLAN program using the MOR and MEGRASP programs (MacWhinney, 2000).

Three raters analyzed 15% of samples to calculate inter-rater reliability. Reliability was calculated using the RELY program within CLAN, and agreement was 92.4% for transcription and 98% for coding of semantic units.

### Discourse Measures

Our discourse measures of interest were derived from a previous study from our group (Mueller et al., 2017), which detected underlying latent factors from multiple discourse measures. The aims of the Mueller et al. (2017) study were the following: (1) to determine the most salient connected language measures for use in this group of asymptomatic, late-middle-aged individuals; (2) to address the problem of multiple comparisons by determining latent constructs to use for analysis rather than many individual measures; (3) to determine the test-retest stability of discourse measures in a group of individuals who were cognitively stable across several study visits and (4) to determine factor invariance across sex and family history of AD. In brief, factor analysis using promax rotation and principal axis factor extraction (Fabrigar et al., 1999) was used to reduce a large set of 18 language measures to a smaller number of latent factors. **Table 1** shows the factors and the connected language measures that loaded on each factor. The factors represent four underlying constructs in discourse: (1) Semantic content refers to the proportion of meaningful content words relative to total words. The Semantic factor is comprised of variables measuring pronouns, verbs and nouns in spontaneous speech; (2) the Syntax factor describes the complexity of sentences, and is represented by measures of grammatical complexity and verb usage within utterances; (3) the Lexical factor describes the diversity of words and is represented by indices of unique words to total words, content units to total words; and the Fluency factor represents the flow of speaking, characterized by an index measuring dysfluencies such as speech repetitions, revisions, and filled and unfilled pauses. For additional information on factor score development, including details on the exploratory and confirmatory factor analyses and factor invariance assessments, see Mueller et al. (2017). We multiplied the Fluency factor and Semantic factors by -1 to standardized directionality across all factor scores, such that negative scores indicated worse performance.

**Table 1:** Latent factor structure, connected language measures and definitions.

Factor name	Connected language measure	Definition
Semantic	Percent nouns	
	Percent verbs	
	Pronoun index	Number of pronouns/nouns + pronouns
Syntax	Verb index	Number of verbs/number of utterances
	Proposition density	Ratio of propositions (verbs, adjectives, adverbs, prepositions and conjunctions) to total number of words, based on Computerized Propositional Idea Density Rater (CPIDER3) (Brown et al., 2008; Covington and McFall, 2010; Macwhinney et al., 2011)
	Grammatical complexity	Number of grammatical relations that mark syntactic embeddings/total number of grammatical relations (Macwhinney et al., 2011)
Lexical	Type-token ratio	Number of unique words/number of words
	Number of unique words	
	Semantic unit idea density (SUID)	Number of semantic units [as defined by Croisile et al. (1996) and Ahmed et al. (2012)]
Fluency	Maze index	Number of filled pauses, false starts, revisions, +repetitions/total number of utterances


### Statistical Analysis

#### Participant Demographics, Clinical Characteristics, Cognitive Functioning, and Baseline Language

Sample characteristics of the cognitively healthy and eMCI groups were compared using *t*-tests for continuous data and chi-square tests for categorical data. Neuropsychological test performance was compared with F statistics, adjusting for age, sex and literacy; in the case of highly skewed data [Mini-Mental State Examination (MMSE), Boston Naming Test (BNT)], we used Mann–Whitney *U*-tests. We correlated participants’ performance on standardized neuropsychological tests with language factors using Spearman’s rank correlation coefficients, due to the skewed distribution of the BNT and MMSE. We examined baseline performance of each of the four connected language factors by cognitive status, adjusting for age, sex and literacy via analysis of covariance (ANCOVA).

#### Relationship between Cognitive Status and Longitudinal Connected Language Performance Trajectories

Analyses were conducted using R version 3.3.2 and SPSS version 22 using linear mixed models to examine longitudinal trends in connected language factor scores and their relation to cognitive status (CH vs. eMCI), the predictor of interest. Linear mixed effects modeling allowed us to examine connected language for participants as a group (fixed effects), while accounting for variation associated with individual differences (random effect of subject-specific intercept) (Laird and Ware, 1982). “Time” was operationalized as age at each visit, in order to account for differences in baseline ages and time intervals between visits. We centered age at the average of 63 years for ease of interpretation, with precision to two decimal places for improved temporal resolution. Additional covariates were included so that fixed effects could vary by sex and literacy [scores on the WRAT-III reading subtest (Wilkinson, 1993)]. Additional covariates examined included: (1) depressive symptoms, as measured by the *Center for Epidemiologic Studies Depression Scale (CES-D)* (Radloff, 1977) total score; (2) anxiety, measured by a binary variable indicating whether or not a participant self-reported a diagnosis of anxiety at either visit; and (3) self-reported sleep quality, measured as a continuous variable, based on the 12-item Rand Sleep Scale Survey used in the Medical Outcomes Study (MOS).

Models were fit using the lme4 package within the R environment (Bates et al., 2014). First, we examined unconditional means and growth models (i.e., subject-level random effects). Growth terms were not significant for any outcome, so random effects used in subsequent models included only subject-specific intercepts. Subsequent models also included fixed effects of sex and literacy. We tested interactions between cognitive status and age, sex, and literacy. Only the cognitive status by age interaction was significant for the Semantic and Fluency factor models; therefore, we used the base models for the Syntax and Lexical factors. As secondary analyses, the final models were re-run with additional fixed effects of depressive symptoms, sleep and anxiety. In each case the final model selected was fit with restricted maximum likelihood estimation (REML).

#### Other Descriptive Connected Language Variables

We extracted connected language variables that have been shown in the literature to be sensitive to MCI or probable AD dementia, but did not load on any of our factors (see Mueller et al., 2017). We performed ANCOVAs adjusting for age, sex, and literacy in order to depict the means at two time points, and mean change, between the CH and eMCI groups.

## Results

### Sample Characteristics

**FIGURE 1:**
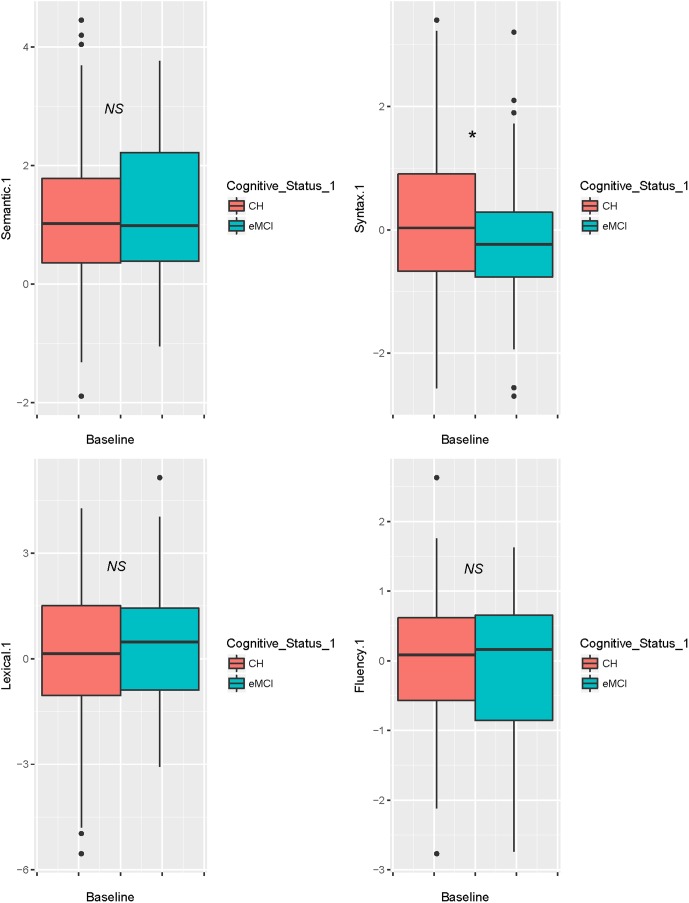
Boxplot showing baseline performance on four connected language factors by cognitive status, adjusting for age, sex, and literacy.

**Table 2** displays sample characteristics by cognitive status (CH, eMCI). **Table 3** presents the raw scores of the neuropsychological tests adjusted for age, sex and literacy (WRAT-III reading). Both tables reflect data collected at the second speech sample visit. Our final sample consisted of data from 264 participants at two time points with a mean of 2.0 years apart (*SD* = 1.5), typically the 4th and 5th wave visits. At the visit with the second speech sample, there were 64 participants classified as eMCI and 200 as CN; 47 of the 64 eMCI participants were also eMCI at the first speech sample. The eMCI group was comprised of significantly more males (54%) and they were slightly older (mean age of 66 at follow-up, versus 64 in the CH group). On average, the groups were similar in terms of years of education, literacy, *APOE*-ε4 status, family history status and depression scores. After adjusting for age, sex and literacy, the two groups differed significantly on tests of memory (RAVLT, Logical Memory-Delayed), executive functions (Digit Symbol, Stroop Interference), and language (Boston Naming Test, category and letter verbal fluency). **Figure 1** shows baseline performance of the four connected language factor scores by cognitive status. Syntax was marginally lower in the eMCI group at baseline (*p* = 0.09).

**Table 2:** Sample demographic and clinical characteristics.

Variable	Total sample	Cognitively healthy at follow-up	EarlyMCI at follow-up	*p-*value
*n*	264	200	64	
eMCI cognitive status at first speech sample		20	28	
Age at first speech sample	61.8 (6.5)	61.1 (6.5)	64.2 (5.9)	**0.04**
Age at second speech sample	64.2 (6.6)	63.6 (6.7)	66.3 (5.9)	**0.04**
Sex (n; %F)	180; 67.4%	141; 70%	36; 56%	**0.03**
Education (y)	16.4 (2.8)	16.5 (2.7)	16.0 (2.8)	0.13
WRAT-III standard score	106.7 (9.3)	106.7 (8.9)	106.7 (10.3)	0.52
*APOE* ε4 allele (n; %ε4 +)	104; 39%	80; 40%	22; 34%	0.49
Parental history of AD (n; %+)	213; 79.8%	159; 80%	52; 81%	0.83
Ethnicity (n; % white)	252; 95.8%	194; 97%	58; 91%	
CES-D		6.6 (0.49)	5.2 (0.77)	0.83
Self-reported hearing loss (n; %+)	9; 3%	7; 4%	2; 3%	0.80


*eMCI, early Mild Cognitive Impairment; F/M, female/male; WRAT-III, Wide Range Achievement Test – Third Edition, reading subtest; *APOE*, apolipoprotein E; AD, Alzheimer’s disease; CES-D, Clinical Evaluation Scale of Depression, MMSE, Mini-Mental State Examination; RAVLT, Rey Auditory Verbal Learning Test, Total score. Items in bold indicate statistically significant difference between CH and eMCI at *p* < 0.05.*

**Table 3:** Neuropsychological test performance at language sample follow-up visit.

Neuropsychological Test	Cognitively healthy at Discourse Baseline	EarlyMCI at Discourse Baseline		Cognitively healthy at follow-up	EarlyMCI at follow-up	

Test			*p-*value	Adjusted Mean^∗^ (*SE*)	Adjusted Mean^∗^ (*SE*)	*p*-value
MMSE^∗∗^	29.5 (0.05)	29.0 (0.17)	0.01	29.5 (0.11)	29.2 (0.12)	0.02
RAVLT – Total	53.0 (0.54)	42.5 (1.0)	<0.001	53.1 (0.62)	41.1 (0.86)	<0.001
Logical Memory Delayed	27.13 (0.45)	20.8 (0.95)	<0.001	27.8 (0.57)	21.3 (0.8)	<0.001
Digit Symbol Coding	57.6 (0.7)	47.8 (1.4)	<0.001	57.2 (0.72)	50.1 (1.3)	<0.001
Stroop – Color-Word	110.1 (1.4)	95.8 (2.8)	<0.001	108.6 (1.5)	100.2 (2.8)	0.009
Boston Naming Test^∗∗^	58.2 (0.17)	56.4 (0.51)	<0.001	58.4 (0.21)	56.6 (0.29)	<0.001
Letter Fluency (CFL)	53.01 (0.53)	42.5 (1.5)	<0.001	51.7 (0.81)	44.6 (1.2)	<0.001
Category Fluency (Animal)	23.5 (0.39)	19.4 (0.55)	<0.001	23.5 (0.35)	20.3 (0.53)	<0.001


*For all tests, higher scores indicate better performance. ^∗^ANCOVA F-statistics were calculated by adjusting means for age, sex, and WRAT-III reading scores.^∗∗^Mann–Whitney *U* tests performed due to highly skewed data.*

**FIGURE 2:**
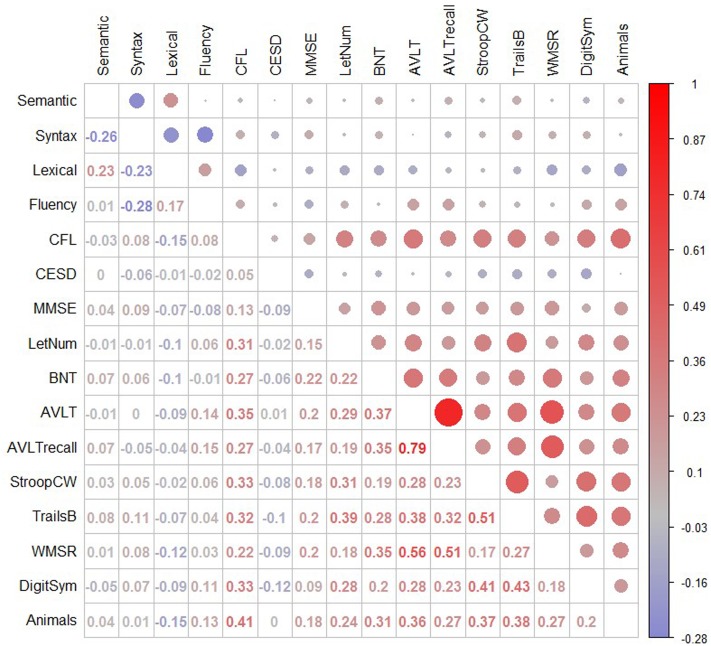
Correlations between language factors and standardized neuropsychological tests. Pearson’s correlation coefficient is shown in the lower diagonal; the upper diagonal depicts magnitude (larger circle = larger correlation) and direction (blue = negative, red = positive). CFL, letter fluency, Controlled Oral Word Association Test (Wechsler, 1987); CESD, Center for Epidemiology Scale for Depression; MMSE, Mini-Mental Status Examination (Folstein et al., 1975); LetNum, Letter Number sequencing from the WAIS-R; BNT, Boston Naming Test from the Boston Diagnostic Aphasia Examination; AVLT, Rey Auditory Visual Learning Test; AVLTrecall, 30 min delayed recall of RAVLT; StroopCW, Color-Word Stroop subtest; Trails B, Total Trails B score (Benton et al., 1994); WMSR, Logical Memory Story Recall from the WMS; DigitSym, Digit Symbol from the WAIS (Wechsler, 1981); Animals, Category fluency.

**Figure 2** depicts correlations between the four connected language factors and select standardized neuropsychological test scores. The Fluency factor was correlated with AVLT delayed recall (*r* = 0.19), AVLT total score (0.18), and Animal Naming (0.14). The Lexical factor was negatively correlated with Letter-Number Sequencing (-0.22), letter fluency (-0.16), BNT (-0.10), Logical Memory delayed recall (-0.12), and Animal Naming (-0.15). Syntax was positively correlated with Trails B (0.11), and the MMSE (0.10). The semantic factor showed a non-significant, weakly positive correlation with the Boston Naming Test (0.08), the RAVLT delayed recall (0.08), and Trails B (0.08).

### Relationships between Cognitive Status and Longitudinal Connected Language Trajectories

Results from linear mixed effects regression models examining the relationships between cognitive status and connected language factor score at each visit are presented in **Table 4**. All four linear mixed effects models were re-run with additional fixed effects of sleep, depression, and anxiety; none of these predictors were significant, thus **Table 4** depicts the simpler models.

**Table 4:** Parameter results from linear mixed effects regression models.

Variable	Semantic		Syntax		Lexical		Fluency	
				
	β (*SE*)	95% CI	β (*SE*)	95% CI	β (*SE*)	95%CI	β (*SE*)	95% CI
Age (centered)	-0.001 (0.01)	-0.017 to 0.015	-0.01 (0.01)	-0.03 to 0.01	-0.02 (0.02)	-0.06 to 0.01	0.01 (0.01)	-0.001 to 0.02
Sex (male)	-0.09 (0.10)	-0.30 to 0.11	-0.24 (0.13)	-0.46 to 0.08	-0.15 (0.24)	-0.62 to 0.32	**0.57 (0.11)^∗∗∗^**	0.36 to 0.78
WRAT-III standard score	-0.28 (0.22)	-0.28 to 0.14	**0.75 (0.27)^∗∗^**	0.23 to 1.3	**-2.7 (0.5)^∗∗∗^**	-0.3.7 to 1.8	-0.70 (0.23)	-1.18 to -0.29
ConsensusDx (eMCI)	-0.07 (0.11)	-0.28 to 0.14	-0.19 (0.14)	-0.46 to 0.07	0.28 (0.23)	-0.2 to 0.72	**-0.23 (0.11)^∗^**	-0.41 to -0.01
Age × Consensus Dx	**-0.04 (0.02)^∗^**	-0.07 to -0.007	—	—	—	—	**-0.03 (0.02)**	-0.06 to -0.001


*SE, standard error; CI, confidence interval; ConsensusDx, consensus diagnosis of either early MCI (eMCI) or cognitively healthy(CH); WRAT-III, Wide Range Achievement Test-3, reading subtest. Items in bold indicate statistically significant effects at ^∗^*p* < 0.05, ^∗∗^*p* < 0.01, ^∗∗∗^*p* < 0.001 without correction for multiple comparisons. No interactions were statistically significant at Bonferroni corrected alpha of *p* < 0.013.*

**FIGURE 3:**
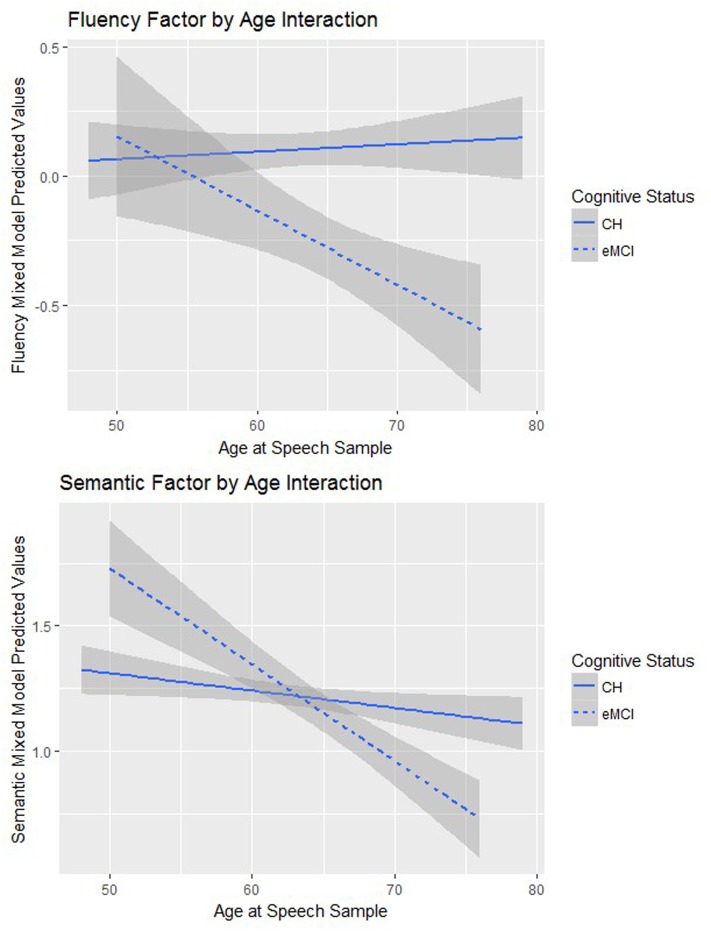
The simple slopes of the interaction between predicted language factor values and age from the linear mixed effects models.

**Figure 3** depicts simple slopes of the interaction between age and cognitive status for mixed model-predicted fluency and semantic values.

#### Semantic Factor

A significant interaction between time (centered age) and cognitive status indicated that individuals who were diagnosed with eMCI declined faster than the CH group on the semantic connected language factor over time (β = -0.04, *p* = 0.03).

#### Syntax Factor

Interactions between age and cognitive status, or cognitive status and the other covariates were not significant; therefore Model 1 was used. Cognitive status was not a significant predictor of syntax performance at either visit.

#### Lexical Factor

No interactions were significant so we retained Model 1. A significant main effect for time for the lexical factor indicated that all individuals tended to decrease in lexical diversity from time one to time two, but cognitive status was not a significant predictor of this decline.

#### Fluency Factor

A significant interaction between time and cognitive status indicated that participants who were diagnosed with eMCI declined faster on fluency factor scores over time (β = -0.03, *p* = 0.03). Cognitive status had a significant main effect on the fluency factor, indicating that eMCI status at either both visits or the most recent visit was a significant predictor of poorer speech fluency scores (β = -0.23, *p* = 0.03).

### Other Descriptive Connected Language Variables

Mean scores and results of ANCOVAs for descriptive connected language variables (that were not part of the factor scores) are presented in **Table 5**. There were significant differences between the eMCI group and the CH group for mean length of utterance (MLU) at both time points (*p* < 0.001), words per minute at Time 2 (*p* = 0.02), and semantic units per minute at Time 2 (*p* = 0.03). Change from Time 1 to Time 2 was not significant between groups for any of the descriptive discourse variables.

**Table 5:** Descriptive variables of language samples at two time points.

	Speech Time 1			Speech Time 2			Change (Time 2-Time 1)		
			
Language variable	CH Mean (*SD*)	eMCI Mean (*SD*)	*p*-value	CH Mean (*SD*)	eMCI Mean (*SD*)	*p*-value	CH Mean (*SD*)	eMCI Mean (*SD*)	*p-*value
Total words	103.4 (47.3)	114.1 (49)	0.41	112 (50)	117 (62.4)	0.52	9.9	8.9	0.92
Total semantic units	13.1 (3.4)	14.2 (2.8)	0.53	14.2 (3.1)	14.3 (2.7)	0.79	1.3	1.0	0.70
MLU	15.7 (12.0)	11.6 (5.9)	**<0.001**	17.7 (14.0)	13.0 (7.3)	** 0.001**	2.0	1.3	0.56
Words per minute	144.0 (32.2)	136.3 (28.7)	0.09	143.3 (28.1)	132.8 (31.7)	**0.02**	-0.27	0.12	0.48
Semantic units per minute	20.2 (7.8)	19.4 (19.4)	0.50	20.6 (8.4)	18.3 (6.3)	**0.03**	0.36	-1.3	0.22


*Items in bold indicate statistically significant difference between CH and eMCI at *p* < 0.05.*

## Discussion

Although a decline in episodic memory is the typical defining feature of AD dementia, changes in language are also evident early in the disease course. Analyses of connected language in the mild to moderate stages of AD dementia has proved to be a sensitive measure of subtle changes in performance, but we did not know if connected language analysis would be similarly sensitive in very early stages of cognitive decline. Thus, we set out to investigate whether connected language performance was associated with a diagnosis of sub-clinical MCI in a risk-enriched cohort of healthy, late-middle-aged individuals.

Our findings suggest that, in this younger and relatively healthy cohort, subclinical declines in memory and/or executive function were associated with changes in connected language. Specifically, as individuals progressed through the study, those who showed eMCI status declined more rapidly than those who were cognitively healthy in both semantic and fluency features of connected language. Lexical diversity and syntactic complexity were not associated with eMCI status in these analyses. When we added sleep quality, depression, and anxiety to the models, the results remained the same and these factors were not significantly associated with language declines.

The literature supports our finding that eMCI participants declined more rapidly than CH participants on semantic measures. Our connected language semantic factor (Mueller et al., 2017) is weighted such that it represents a higher proportion of pronouns, a lower percentage of nouns, and an increase in use of verbs. Studies using composite scores or factor scores similar to our semantic factor (Mueller et al., 2017) have found comparable results later on the AD continuum. For example, Fraser et al. (2016) trained a machine learning classifier on 370 linguistic features of Cookie Theft picture descriptions from 167 adults with AD and 97 controls. They subsequently performed a factor analysis of successfully classifying features, which resulted in a semantic factor that included of a high proportion of pronouns, low proportion of nouns, and high verb frequency (i.e., less semantic content overall). This semantic factor differentiated speech samples of adults with AD from those of controls (Fraser et al., 2016). Ahmed et al. (2013b) used a composite score comprised of measures of pronoun ratios and verb ratios, which they termed “lexical content,” to examine 9 adults across three stages of autopsy-confirmed AD: MCI, mild AD, and moderate AD. Composite scores differentiated between MCI and mild AD stages, but trends in the difference between controls and patients with MCI did not reach statistical significance (Ahmed et al., 2013b). Our study sample may represent a stage that is even earlier on the AD continuum than MCI, and the finding of significant differences in our group is likely because of our large sample size.

The higher loading of verbs relative to nouns in our semantic factor is a phenomenon that has been explored previously in language of adults with AD. There is an emerging literature regarding putative neuroanatomical substrates of verb vs. noun storage (Damasio and Tranel, 1993; Daniele et al., 1994). Future research involving network or regional specificity to our findings may help to further understand these results.

With respect to high pronoun index, Almor et al. (1999) showed that adults with AD (*n* = 11) used more pronouns in relation to noun phrases than controls (*n* = 9) (Almor et al., 1999). While several other studies of adults with AD report a similar over-use of pronouns in connected language (Ripich and Terrell, 1988; Ulatowska et al., 1988), such findings in studies of MCI are limited. Our study adds evidence to the notion that connected language may become semantically impoverished early on the continuum of cognitive decline.

Correlations between the semantic factor and standardized measures of semantic content (BNT and animal naming) were not statistically significant. This finding is partially supported by the connected language literature, but results of that literature are mixed. Almor et al. (1999) found no correlation between increased pronoun use and picture naming scores, and theorized that pronoun measures in discourse may be more a reflection of working memory problems than of semantic memory. Nicholas et al. (1985) similarly found no correlation between discourse measures of “empty speech” (e.g., pronouns without referents) and scores on picture naming tasks. Kavé and Goral (2016) found that picture naming and semantic measures of Cookie Theft descriptions were correlated in a group of adults with AD (*n* = 20; *r* = 0.46–0.67), but not in a control group. Kavé and Goral (2016) also showed that semantic verbal fluency scores were not correlated with semantic measures of connected language in either the group with AD or the controls. Moreover, in a longitudinal study of cognitive decline (6–10 years follow-up), Hodges et al. (2006) found that scores in category fluency were among the first to decline, while the onset of other semantically based deficits varied from one to 6 years. In present study, the eMCI group differed significantly from the CH group on verbal fluency and picture naming tasks; the lack of correlation of these measures with discourse measures lends support to the idea that measures of connected language describe not only semantic knowledge and expression, but also the coordination of multiple cognitive processes and are independent of traditional clinically administered language tests. Further, the results of these correlations may change over time as participants age and experience more significant cognitive impairment.

Discourse fluency declined more rapidly in persons with eMCI, and that fluency change was a predictor of eMCI status. A few studies of MCI have shown similar results; for example, Berisha et al. (2015) found that former President Ronald Reagan’s unscripted speeches contained more filled pauses over time (e.g., “um,” “well”) during a period of 6–13 years prior to his diagnosis of AD. By contrast, discourse fluency was not a distinguishing factor in MCI, mild, or moderate AD in the 9 adults studied by Ahmed et al. (2013b), although this sample was very small. Other studies of connected language in AD dementia have shown increased repetitions and revisions (Nicholas et al., 1985; Tomoeda and Bayles, 1993), increased pauses or hesitations (Davis and Maclagan, 2009; Hoffmann et al., 2010; Gayraud et al., 2011; Sajjadi et al., 2012), and an increase in repaired errors (McNamara et al., 1992). It is possible that these mixed results with fluency measures are due to examining only select disfluent behaviors. Our fluency factor consisted of an index that included filled and unfilled pauses, repetitions, revisions, and false starts divided by the number of utterances. To our knowledge, no studies of discourse in MCI or AD dementia have examined a comprehensive fluency measure such as this, and it is possible that a ratio of fluency behaviors is more sensitive than the individual measures alone. Further, while semantic deficits are common in connected language of people with AD dementia, it is possible that at very early stages a surge in dysfluent behavior may reflect the beginnings of difficulty with word search and retrieval, foreshadowing more problems expressing semantic content. There is evidence that error monitoring does not decline until later in the disease (McNamara et al., 1992; Forbes et al., 2001); so perhaps adults with eMCI in our study noticed and repaired more errors (hence had a higher number of “revisions”), resulting in higher dysfluency scores.

Correlations between the fluency factor and standardized neuropsychological test scores were weakly positive with semantic fluency, AVLT total score, and AVLT delayed recall. These data suggest that dysfluent behavior in the eMCI group may be related to aspects of memory (semantic storage of words) (Henry et al., 2004), executive functions (search and retrieval of words) (Alvarez and Emory, 2006), and working memory (Almor et al., 1999; Hartsuiker and Kolk, 2001).

While syntax is commonly described as “intact” until more severe stages of AD, declines and differences in syntax *complexity* have been observed at mild and moderate stages (Croisile et al., 1996; Kemper et al., 2001). In our sample, syntactic complexity was not associated with eMCI status. Our syntax measure is comprised of a verb index (ratio of verbs to utterances), a grammatical complexity measure based on embedded clauses (Macwhinney et al., 2011), and a measure of propositional density. Snowden et al. (Snowdon et al., 1996) examined the early life free writing samples of cloistered nuns and found that lower idea density at an average age of 22 significantly increased the risk of AD 58 years later. Kemper et al. (2001) examined Nun Study data over time and found that those who met criteria for dementia later in life had lower baseline measures of idea density and grammatical complexity. These results have been explained by both the *cognitive reserve hypothesis* and the *early neuropathology hypothesis*. According to the cognitive reserve hypothesis, nuns with high linguistic abilities either had AD-type neuropathology but were able to either compensate for changes because of their high linguistic capacity. In both models we tested (logistic and linear mixed models), literacy was significantly associated with syntax, which lends some support to the cognitive reserve hypothesis. Conversely, Snowdon et al. (1996) proposed that low linguistic ability early in life might have signaled that development of neuropathology had already begun, decades before dementia was expressed. Comparing baseline syntax measures to longitudinal, multi-modal measures of amyloid deposition and tau protein formation via cerebral spinal fluid and imaging may help to clarify these associations in WRAP participants in the future.

Our lexical factor was not associated with eMCI status, which was surprising given evidence of decreases in lexical diversity and efficiency in adults with AD and MCI (Ahmed et al., 2013b; Fraser et al., 2016). The lexical factor was comprised of measures of lexical diversity (type-token ratio) and semantic unit idea density. That the lexical factor was negatively correlated with phonemic fluency was also unexpected. We examined several transcripts of those with low measures on the lexical factor and found that these picture descriptions contained an overabundance of words with fewer expression of semantic units (i.e., verbosity), as we expected, which would suggest a link between lexical aspects of discourse and eMCI status. It is possible that our lack of significant findings was related to the variable length of transcripts, as type-token ratio is influenced by sample size. One small study of 8 people with MCI and 14 controls found that the MCI group showed significantly higher “potential vocabulary size” (an adapted type-token ratio) than the controls (Aramaki et al., 2016). The authors argued that the adults with MCI may have been compensating for cognitive decline by talking more without necessarily conveying more content. An adapted type-token ratio might be more revealing in future studies, as would use of a task that elicited longer language samples.

We examined other metrics of the language samples that were not included in the factor structure, but were important to include as descriptors, such as total words and words per minute. We also examined MLU, as some studies have shown shorter utterance length for individuals with MCI and AD (Ripich et al., 2000; Ahmed et al., 2013b). We found that the eMCI group had significantly shorter utterances (MLU) at both time points than the CH group. The groups did not differ on total number of words or total number of semantic units. The fact that semantic units were similar for both groups at both time points is surprising, since previous studies of preclinical AD have shown this measure to differentiate between groups (Cuetos et al., 2007; Ahmed et al., 2013a; Mueller et al., 2016). Both groups showed an increase in total words from Time 1 to Time 2, so it is possible that talking more also allowed for more elements of the picture to be described. That the eMCI group expressed fewer words per minute and semantic units per minute indicates that the efficiency of descriptions was compromised, and the time it takes to convey content is a more sensitive measure at this stage.

Sleep, depression and anxiety were not associated with declines in connected language. Murray (2010) examined discourse variables in adults with clinical depression, mild AD, and adults with no psychiatric or neurological diagnosis. While adults with AD produced less informative discourse, adults with depression and control groups did not differ on any discourse variable. Although poor sleep quality has been associated with cognitive decline (Lim et al., 2013), no such associations have been reported with connected language. However, sustained speech analysis was able to detect participants with obstructive sleep apnea (OSA) with 89% accuracy (Blanco et al., 2013), so it is possible that a more detailed speech and voice analysis may yield more information about the relationship between sleep quality and discourse features. Anxiety has been associated with increases in dysfluency in persons who stutter (Yang et al., 2017), and with pragmatic language difficulties (Cummings, 2007), but research is lacking regarding the relationship between anxiety and connected language in the general population or in persons with cognitive decline. As our WRAP sample ages and we continue to collect discourse samples, we will be able to examine these relationships with additional data points.

Our study had strengths and limitations. A strength was the sample size, which far exceeded that of any other prospective longitudinal study of connected language in older adults at risk for AD. In addition, by using a confirmed structure of latent factors, we were able to summarize measures in broad categories of language performance and better characterize our results. The standardized and focused nature of the picture description task was another strength, as it minimized demands on episodic memory, but still yielded spontaneous language. The Computerized Language Analysis program (CLAN) also was a benefit, as it provided a quick, efficient, objective, and standardized means of extracting data; this combined with our relatively simple factor structure and the ubiquity of the Cookie Theft picture in the AD literature will support replication of our analysis in future studies.

One of the limitations of our study was the relatively short length of the language samples (average total words = 109). While there is no established minimum length for language samples in adults with MCI, for adults with non-fluent aphasia, Brookshire and Nicholas recommended an average of 300–400 words (Brookshire and Nicholas, 1994), and others recommended a minimum of 150 words (Saffran et al., 1989; Sajjadi et al., 2012). Longer samples might be needed for individuals with non-fluent aphasia, however, given their reduced language output. Fraser et al. (2016) also had a mean sample length of 100 words from patients with AD, and reported findings with some parallels to ours, so 100 words may be sufficient. The short task may also be considered a strength, as it presents a relatively low burden for participants compared to demands of typical standardized tests. If subtle changes can be detected in shorter samples, this will save time for both the participant and the clinician. Future longitudinal research in order to develop sample size recommendations in this group would be beneficial. An additional limitation includes that we did not adjust for multiple comparisons when examining the four linear mixed effects models (*p* = 0.03 vs. Bonferonni-adjusted criteria of *p <* 0.013). Future research will address this limitation as our sample size of transcribed language samples increases, and as additional time points become available.

Although our approach proved to be feasible in this large cohort, the transcription and coding of these language samples was relatively labor-intensive. The field of computational linguistics has made advances in machine learning techniques for analyzing linguistic features, but these methods also require transcriptions of spoken language. Researchers are beginning to improve upon Automatic Speech Recognition (ASR) to solve this problem, which will allow for more efficient analyses that can be translated to clinical practice for disease monitoring.

A limitation exists within the WRAP sample itself: it is predominantly white, well-educated, and primarily from the Upper Midwest region of the United States. Future projects should validate these results in more diverse cohorts.

Finally, while the very early, sub-clinical construct of eMCI can be considered a strength since no other studies have examined connected language in an at-risk cohort, the participants diagnosed with eMCI may have an etiology that is not AD. Thus one cannot conclude from these results alone that our method of connected language analysis would be a sensitive tool for detecting decline in persons with known preclinical AD, which in research can now be characterized by the presence of beta-amyloid, tau protein accumulation, and neurodegeneration, evidenced by PET-imaging, cerebrospinal fluid analysis, and MRI (Jack et al., 2016). Further, the term ‘early’ in the context of this work simply connotes cognitive decline of less severity than MCI and does not refer to any age of onset specifically. Future research will address this limitation by taking these analyses further and examining connected language in individuals with preclinical AD based on biomarker evidence of AD pathology. We also plan to continue to follow these participants and collect future language samples and biomarkers in order to determine the course of connected language change as the WRAP cohort ages.

## Conclusion

In a review of assessment in AD and dementia, Laske et al. (2015) listed connected language analysis as one of the most promising state-of-the-art diagnostic measures for MCI and AD. The authors described spoken language as a multi-dimensional, non-invasive, and informative biological sample for the early detection of AD, primary progressive aphasia, and other dementia syndromes. Our results provide evidence that features of connected language are associated with very early, sub-clinical declines in memory in late-middle age. This research contributes toward a better understanding of early language changes in conjunction with cognitive decline. Future validation of this method in a preclinical AD sample may show that connected language analysis may be a candidate tool for only improve disease monitoring in clinical trials, but also for informing cognitive-communication interventions and caregiver education programs that maintain and enhance quality of life and life participation for adults with MCI and AD dementia.

## Author Contributions

KM and LT conceived the idea of the work. KM, LT, RK, BH, and SJ contributed to the statistical plan and study design, and the analysis and interpretation of data for the work. KM drafted the work, and LT, RK, BH, and SJ revised it critically for important intellectual content. KM, LT, RK, BH, and SJ provided final approval of the version to be published. KM, LT, RK, BH, and SJ provided agreement to be accountable for all aspects of the work in ensuring that questions related to the accuracy or integrity of any part of the work are appropriately investigated and resolved.

## Conflict of Interest Statement

The authors declare that the research was conducted in the absence of any commercial or financial relationships that could be construed as a potential conflict of interest.
